# CA724 predicts overall survival in locally advanced gastric cancer patients with neoadjuvant chemotherapy

**DOI:** 10.1186/s12885-020-07666-8

**Published:** 2021-01-05

**Authors:** Yilin Tong, Yan Zhao, Zexing Shan, Jianjun Zhang

**Affiliations:** Department of Gastric Surgery, Liaoning Cancer Hospital and Institute, Cancer Hospital of China Medical University, No 44 of Xiaoheyan Road, Dadong District, Liaoning 110042 Shenyang, China

**Keywords:** Gastric cancer, Tumor marker, Neoadjuvant therapy, CA199, CA125, CA724

## Abstract

**Background:**

Serum tumor markers including AFU, AFP, CEA, CA199, CA125 and CA724, are of great importance in the diagnosis, prognostic prediction and recurrence monitoring of gastrointestinal malignancies. However, their significance in gastric cancer (GC) patients with neoadjuvant therapy (NCT) is still uncertain. The aim of this study was to evaluate the predictive value of these six tumor markers in locally advanced GC patients who underwent NCT and curative surgery.

**Methods:**

In total, 290 locally advanced GC patients who underwent NCT and D2 radical gastrectomy were retrospectively analyzed. Data on their tumor markers before (pre-) and after (post-) NCT and pathological characteristics were extracted from the database of our hospital. The optimal cutoff values of the six tumor markers were calculated by the ROC curve and Youden index. Their predictive significance was analyzed and survival curves for overall survival (OS) were obtained by the Kaplan-Meier method. Associations between categorical variables were explored by the chi-square test or Fisher’s exact test. Multivariate analyses were performed by the Cox regression model.

**Results:**

Pre- and post-CA199, −CA125 and -CA724 could predict overall survival (all *P* < 0.05), but only the change (diff-) of CA199 was related to prognosis (*P* = 0.05). In the multivariable analysis, pre- (*P* = 0.014) and post-CA724 (*P* = 0.036) remained significant, though diff-CA724 was not an independent prognostic factor (*P* = 0.581). In addition, pre- and post-CA199, −CA125 and -CA724 were associated with lymph node metastasis (N- vs N+) and pathological stage (I-II vs III) (all *P* < 0.05). Moreover, post-CA724 was related to the vascular or lymphatic invasion (*P* = 0.019), while pre-CA724 was not (*P* = 0.082). However, AFU, AFP and CEA showed no association with survival (*P* > 0.05).

**Conclusions:**

CA724 is an independent factor for prognosis and could be used to predict ypN and ypTNM stage in locally advanced GC patients undergoing NCT and curative resection.

## Background

Gastric cancer (GC) is the fifth most frequently diagnosed cancer and the third leading cause of cancer death worldwide [1]. Excellent outcomes could be expected from surgery alone when gastric cancer is diagnosed at an early stage. However, in locally advanced GC patients, surgery does not always lead to a satisfactory outcome, even with postoperative therapy [2]. Neoadjuvant chemotherapy (NCT) improves the R0 resection rate and prognosis when compared with surgery alone or surgery with postoperative therapy [3], but the outcomes are vary due to the differences in many factors such as tumor differentiation and Lauren classification. More indicators to assess survival are urgently needed.

Serum tumor markers play important roles in the diagnosis, prognostic prediction and recurrence monitoring of gastrointestinal malignancies. As studies have suggested, AFU was considered to be related to liver metastasis in colorectal cancer [4]; AFP was associated with prognosis in gastric cancer patients undergoing surgery alone [5]; preoperative CEA could predict the prognosis of GC patients with no lymph node metastasis [6]; CA199 was an independent prognostic factor in gastroesophageal junction (GEJ) cancer patients who experienced surgery alone [7]; the CA125 level was related to the degree of peritoneal dissemination and the existence of malignant ascites in GC patients with peritoneal metastasis [8]; and CA724 was correlated with pTNM stage in gastric carcinoma patients [9]. However, for GC patients who underwent NCT, the evidence of these markers is still insufficient.

In this article, we investigated the prognostic significance of the six serum tumor markers before (pre-) and after (post-) NCT, the predictive value of changes (diff-) of tumor markers due to treatment, the inner-relationships among those markers and the connections between markers and other pathological factors in locally advanced GC patients.

## Methods

Between June 2010 and July 2016, patients with locally advanced gastric adenocarcinoma (including gastroesophageal junction carcinoma) who underwent preoperative chemotherapy with or without postoperative treatment were identified from the database of our hospital. The inclusion criteria were as follows: (1) histopathological evidence of gastric adenocarcinoma; (2) locally advanced tumor (8th edition AJCC clinical staging II-III, T2N1M0-T4N3M0); (3) underwent NCT with or without postoperative treatment; and (4) underwent curative gastrectomy with D2 lymph node dissection. Patients who underwent preoperative radiotherapy, or suffered from other malignant tumors were excluded.

The levels of pre- and post-NCT serum tumor markers including AFU, AFP, CEA, CA199, CA125 and CA724, and other clinicopathological characteristics of all patients were extracted from our database.

The optimal cutoff values of all serum tumor markers and other continuous variables were calculated by ROC curves and the Youden index. The relationships of classified variables were computed by the chi-square test or Fisher’s exact test, and intensities of association were evaluated by the coefficient of contingency (C value). Survival curves for overall survival (OS) were obtained using the Kaplan-Meier method, and the log-rank test was used to compare survival differences. Cox regression analysis was used to assess the hazard ratios of all factors for OS, and the factors with a *P* value ≤0.05 or with great importance in clinical diagnosis were included in the multivariable analysis. OS was calculated as the time from the initial treatment to death by any cause or the last follow-up. The data were analyzed with SPSS 25.0 software.

## Results

### Patient characteristics

From a total of 3196 patients, 290 patients met the inclusion criteria. Their clinicopathological features are shown in Table 1. There were 215 males (74.1%) and 75 females (25.9%), with an age range of 25–77 years (median 59 years). Regarding the tumor location, a majority of tumors were located in the lower third part of the stomach (59.3%), while 8 (2.8%) were located in the GEJ, and 24 (8.3%) were in the diffuse group. Most patients underwent preoperative therapy with SOX (73.8%), and the median number of NCT cycle was 2 (range from 2 to 4). The median operation interval, the time from the end of neoadjuvant treatment to the surgery, was 4 weeks (range from 1 to 9). Thirty-one (10.7%) patients did not receive postoperative treatment. The median follow-up time of all patients was 41 months (range from 3 to 91 months).

**Table 1:** Clinicopathological characteristics

Characteristics	No. of patients	Percent
Gender		
Male	215	74.1
Female	75	25.9
Age		
<65	221	76.2
≥ 65	69	23.8
Blood type		
A	101	34.8
B	73	25.2
AB	28	9.7
O	88	30.3
Smoking		
No	128	44.1
Yes	162	55.9
Drinking		
No	194	66.9
Yes	96	33.1
Family history		
No	230	79.3
Yes	60	20.7
Tumor location		
GEJ	8	2.8
Upper third	32	11.0
Middle third	54	18.6
Lower third	172	59.3
Diffuse	24	8.3
Tumor size (cm)		
< 5	115	39.7
≥ 5	175	60.3
ypT		
0	9	3.1
1–2	57	19.6
3–4	224	77.3
ypN		
0	100	34.5
1	49	16.9
2	79	27.2
3	62	21.4
ypTNM		
I	52	17.9
II	71	24.5
III	167	57.6
Histological type		
Adenocarcinoma	186	64.1
Poorly cohesive carcinoma	104	35.9
Lauren classification		
Intestinal	143	49.3
Diffuse or Mixed	147	50.7
Grade of differentiation		
Well	70	25.2
Moderate or Poor	220	74.8
Vascular or lymphatic invasion		
No	218	75.2
Yes	72	24.8
Nervous invasion		
No	222	76.6
Yes	68	23.4
Neoadjuvant therapy		
SOX	214	73.8
XELOX	21	7.2
FOLFOX	55	19.0
Adjuvant treatment		
No	31	10.7
Yes	259	89.3

*GEJ* Gastroesophageal junction

Pathologically, the average number of removed lymph nodes was 27, and 100 (34.5%) patients had no lymph node metastasis. Nearly half of the tumors (49.3%) were the intestinal type, and only one-fourth (25.2%) were well differentiated. Patients with vascular or lymphatic invasion (VOLI) accounted for 24.8%, while those with nerve invasion (NI) accounted for 23.4%.

### Prognostic significance of serum tumor markers

Because of the nature of retrospective studies, not all patients had data on the six serum tumor markers. The number of patients whose markers were available before and after NCT are shown in Additional file 1. The levels of pre- serum tumor markers were measured within 4 weeks before the beginning of NCT, and the post- serum levels were measured within 2 weeks before gastrectomy.

**Fig. 1:**
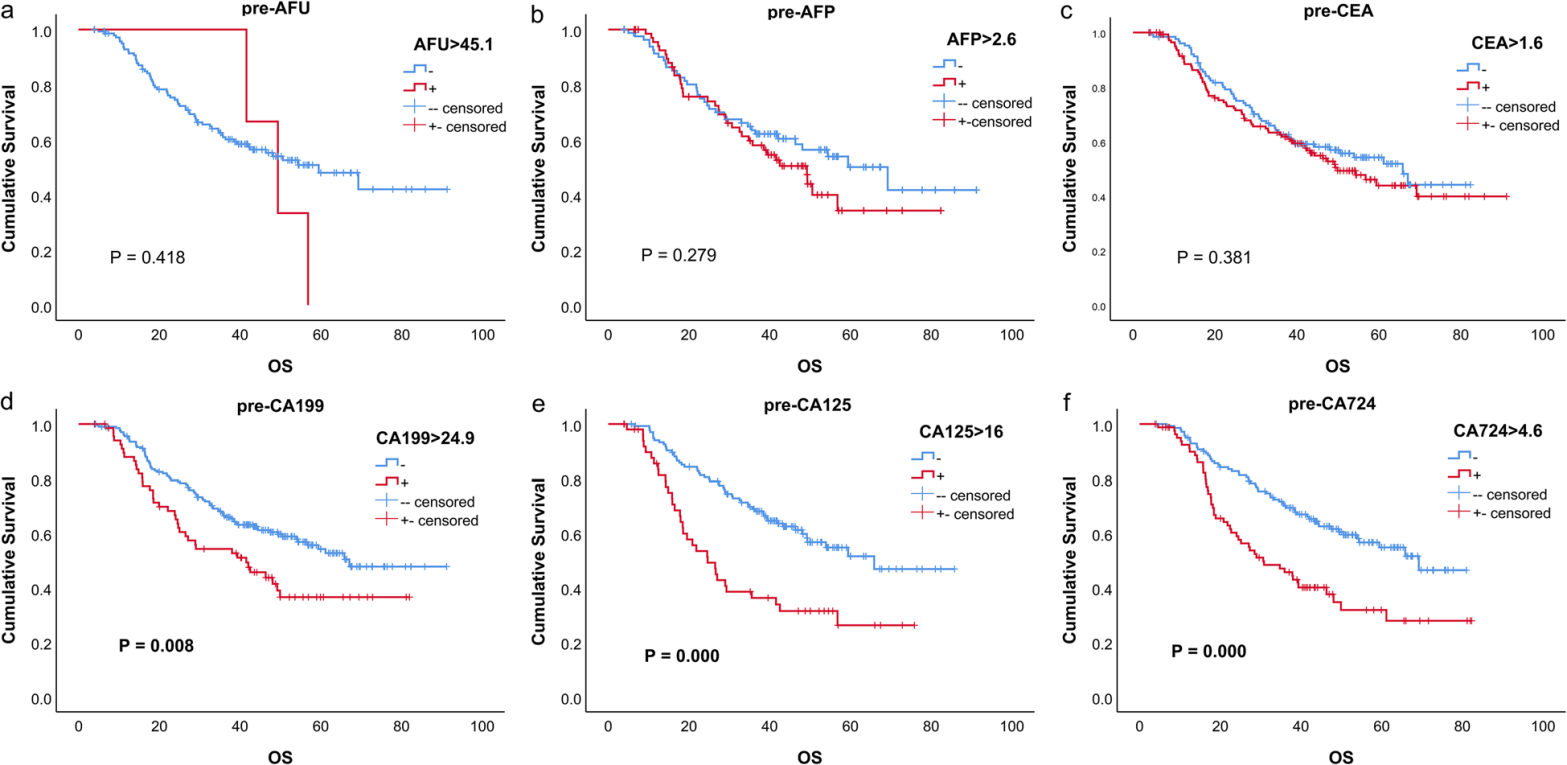
Kaplan–Meier curves of six tumor markers before neoadjuvant therapy

**Fig. 2:**
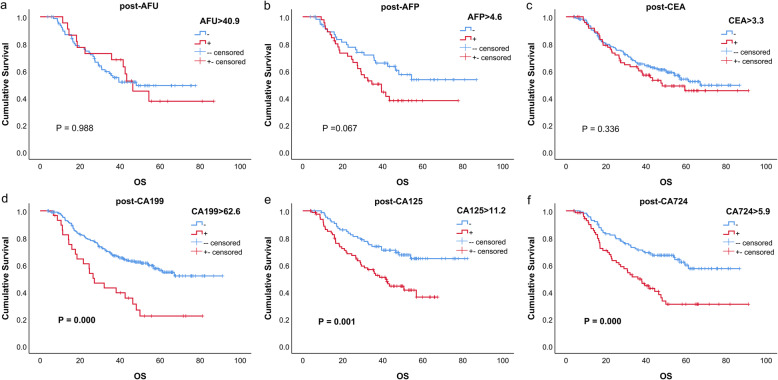
Kaplan–Meier curves of six tumor markers after neoadjuvant therapy

**Fig. 3:**
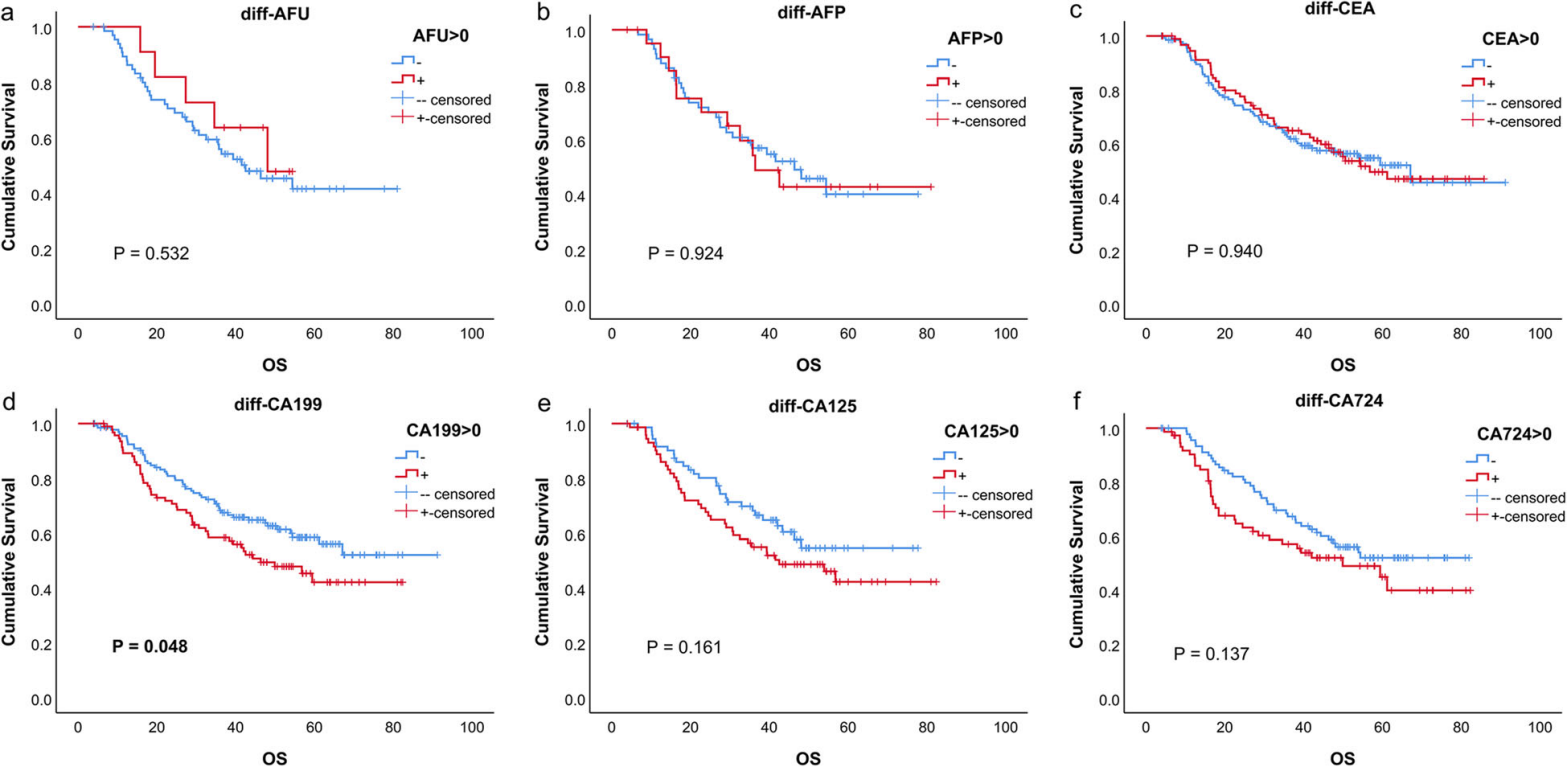
Kaplan–Meier curves of the change of six tumor markers due to neoadjuvant therapy

The univariate analysis outcomes of every marker are listed in Table 2, and the survival curves are shown in Figs. 1, 2, and 3. These results indicated that for CA199, CA125 and CA724, all positive groups had a worse prognosis (all *P* < 0.05). For AFU, AFP and CEA, no significant difference was found (all *P* > 0.05).

**Table 2:** Univariate analysis of tumor markers

Tumor marker	Hazard ratio (95% CI)	*P* value
pre-AFU > 45.1	1.607 (0.504, 5.126)	0.423
pre-AFP > 2.6	1.294 (0.810, 2.066)	0.280
pre-CEA > 1.6	1.193 (0.841, 1.893)	0.322
pre-CA199 > 24.9	1.729 (1.187, 2.519)	**0.004**
pre-CA125 > 16	2.337 (1.515, 3.606)	**0.000**
pre-CA724 > 4.6	2.033 (1.391, 2.972)	**0.000**
post-AFU > 40.9	0.995 (0.517, 1.915)	0.988
post-AFP > 4.6	1.697 (0.958, 3.005)	0.070
post-CEA > 3.3	1.220 (0.813, 1.831)	0.337
post-CA199 > 62.6	2.447 (1.515, 3.954)	**0.000**
post-CA125 > 11.2	2.187 (1.352, 3.536)	**0.001**
post-CA724 > 5.9	2.246 (1.460, 3.455)	**0.000**
diff-AFU > 0	0.742 (0.290, 1.899)	0.533
diff-AFP > 0	1.017 (0.718, 1.439)	0.924
diff-CEA > 0	0.992 (0.814, 1.120)	0.940
diff-CA199 > 0	1.217 (1.000, 1.481)	**0.050**
diff-CA125 > 0	1.190 (0.932, 1.520)	0.163
diff-CA724 > 0	1.186 (0.946, 1.486)	0.139

Units: AFU(U/L); AFP (ng/ml); CEA (ng/ml); CA199(U/ml); CA125(U/ml); CA724(U/ml)

Pre-, post-, and diff-CEA, −CA199, −CA125 and -CA724 were included in the multivariate analysis (Table 3). In the pre- and post- groups, CA724 was an independent prognostic factor, while other markers did not. However, in diff- group, CA724 lost its predicting value (*P* = 0.581), though CA125 was an independent prognostic factor (*P* = 0.034).

**Table 3:** Multivariate analysis of all characteristics on overall survival

Variable	pre-HR (95% CI)	*P* value	post-HR (95% CI)	*P* value	diff-HR (95% CI)	*P* value
Age	1.049 (0.565, 1.948)	0.879	0.569 (0.254, 1.272)	0.169	0.556 (0.242, 1.277)	0.166
Tumor Location		0.160^*^		0.020^*^		0.318^*^
Upper third	1		1		1	
GEJ	0.298 (0.048, 1.849)	0.194	0.470 (0.060, 3.698)	0.473	0.314 (0.025, 3.883)	0.367
Middle third	0.512 (0.186, 1.406)	0.194	0.108 (0.020, 0.586)	**0.010**	0.192 (0.031, 1.205)	0.078
Lower third	0.532 (0.222, 1.278)	0.158	0.265 (0.083, 0.844)	**0.025**	0.542 (0.163, 1.801)	0.318
Diffuse	1.170 (0.420, 3.259)	0.763	0.872 (0.261, 2.912)	0.824	0.876 (0.216, 3.556)	0.853
Tumor Depth (cm)	1.493 (0.738, 3.023)	0.265	1.695 (0.615, 4.669)	0.308	1.655 (0.610, 4.488)	0.322
ypT		0.425^*^		0.051^*^		0.109^*^
0	1		1		1	
1–2	0.212 (0.016, 2.860)	0.243	0.103 (0.005, 2.333)	0.153	0.129 (0.006, 2.933)	0.199
3–4	0.414 (0.022, 7.627)	0.553	1.207 (0.020, 71.782)	0.928	1.018 (0.019, 55.965)	0.993
ypN		0.036^*^		0.003^*^		0.004^*^
0	1		1		1	
1	6.372 (1.280, 31.734)	**0.024**	45.419 (6.213, 332.002)	**0.000**	26.013 (3.647, 185.553)	**0.001**
2	7.865 (1.399, 44.214)	**0.019**	36.926 (4.488, 303.797)	**0.001**	21.381 (2.539, 180.016)	**0.005**
3	13.512 (2.217, 82.368)	**0.005**	49.034 (5.063, 474.881)	**0.001**	52.701 (5.358, 518.388)	**0.001**
ypTNM		0.759^*^		0.192^*^		0.256^*^
I	1		1		1	
II	1.449 (0.209, 10.075)	0.708	0.582 (0.031, 10.892)	0.718	0.571 (0.028, 11.584)	0.716
III	0.927 (0.059, 14.55)	0.957	0.103 (0.002, 5.181)	0.256	0.114 (0.002, 5.424)	0.271
Histological type	0.583 (0.309, 1.097)	0.094	1.234 (0.562, 2.710)	0.600	1.168 (0.515, 2.648)	0.710
Lauren Classification	1.774 (0.972, 3.238)	0.062	0.919 (0.426, 1.982)	0.830	1.022 (0.422, 2.476)	0.961
Grade of differentiation	2.592 (1.074, 6.257)	**0.034**	3.495 (0.980, 12.457)	0.054	4.807 (1.345, 17.176)	**0.016**
VOLI	1.623 (0.925, 2.849)	0.091	4.532 (2.045, 10.043)	**0.000**	2.529 (1.005, 6.364)	**0.049**
NI	0.946 (0.543, 1.646)	0.843	0.957 (0.501, 1.829)	0.894	1.120 (0.544, 2.305)	0.758
Adjuvant therapy	3.421 (1.488, 7.866)	**0.004**	5.853 (1.919, 17.852)	**0.002**	7.236 (2.419, 21.647)	**0.000**
CEA	1.265 (0.712, 2.247)	0.423	1.105 (0.511, 2.390)	0.800	0.903 (0.399, 2.044)	0.807
CA199	0.984 (0.530, 1.829)	0.960	1.570 (0.606, 4.066)	0.353	1.032 (0.483, 2.206)	0.936
CA125	1.503 (0.821, 2.754)	0.187	1.492 (0.774, 2.878)	0.232	2.353 (1.068, 5.184)	**0.034**
CA724	2.177 (1.169, 4.056)	**0.014**	2.349 (1.057, 5.220)	**0.036**	1.238 (0.581, 2.638)	0.581

*VOLI* Vascular or lymphatic invasion, *NI* Nervous invasion, *GEJ* Gastroesophageal junction; *P values from joint tests

### Correlation within serum tumor markers

Because the significance of the tumor markers except for CA724 was lost in multivariate analysis, we analyzed the interconnections between these markers. Their coefficients of contingency are shown in Table 4. In the pre- group, the positive rates of CA199 (*P* = 0.005) and CA125 (*P* = 0.015) were significantly higher when CEA was positive, and the positive rates of CA125 (*P* = 0.001) and CA724 (*P* = 0.002) were significantly higher when CA199 was positive. However, for the post- indicators, only the relation between CEA and CA125 was still remarkable (*P* = 0.014). Associations were also found in the diff-group, although the correlations among all tumor markers were not very strong (all C values < 0.3).

**Table 4:** Coefficient of contingency (C value) among tumor markers

Markers				C value	*P* value
pre-CEA		–	+		
pre-CA199	–	104	96	0.169	**0.005**
	+	22	46		
pre-CA125	–	71	62	0.177	**0.015**
	+	17	34		
pre-CA199		–	+		
pre-CA125	–	109	27	0.230	**0.001**
	+	29	22		
pre-CA724	–	115	25	0.205	**0.002**
	+	52	30		
post-CEA		–	+		
post-CA199	–	152	57	0.125	0.052
	+	16	13		
post-CA125	–	67	20	0.185	**0.014**
	+	50	34		
post-CA724	–	85	27	0.133	0.069
	+	45	26		
post-CA199		–	+		
post-CA125	–	82	5	0.130	0.089
	+	71	11		
diff-CEA		–	+		
diff-CA199	–	45	47	0.150	**0.024**
	+	44	86		
diff-CA125	–	32	41	0.136	0.098
	+	22	50		
diff-CA199		–	+		
diff-CA125	–	40	32	0.165	**0.045**
	+	28	44		
diff-CA724	–	36	36	0.203	**0.008**
	+	27	64		

Only results with *P* ≤ 0.1 were listed

### Relationship between the serum tumor markers and pathological factors

The correlations between the markers and pathological factors were analyzed (Table 5). The lymph node metastasis rates were significantly higher in the positive groups of pre- and post-CA199, −CA125 and -CA724. In the diff- category, no correlation was found. The increase of pre- and post-CA199 and CA125 could predict a worse ypTNM stage (all *P* < 0.05). Although pre-CA724 owned a similar function (*P* = 0.007), the post- serum level lost its ability to predict the pathological stage (*P* = 0.098). In terms of VOLI, only post-CA724 was related to the invasion rate (*P* = 0.019). In addition, there was no correlation between the markers and NI.

**Table 5:** Relationship between serum tumor markers and pathological factors

		N stage		*P* value	ypTNM		*P* value	VOLI		*P* value
		N-	N+		I-II	III		+	–	
pre-CA199	–	80	125	**0.009**	93	112	**0.050**	156	49	0.400
	+	15	54		22	47		49	20	
pre-CA125	–	62	74	**0.000**	69	67	**0.001**	102	34	0.116
	+	6	46		12	40		33	19	
pre-CA724	–	49	92	**0.039**	63	78	**0.007**	110	31	0.082
	+	18	65		22	61		56	27	
post-CA199	–	81	133	**0.003**	100	114	**0.003**	159	55	0.559
	+	3	26		5	24		23	6	
post-CA125	–	44	44	**0.001**	49	39	**0.014**	65	23	0.854
	+	22	62		31	53		61	23	
post-CA724	–	45	69	**0.022**	53	61	0.098	91	23	**0.019**
	+	17	56		25	48		47	26	
diff-CA199	–	34	62	0.833	41	55	0.969	70	26	0.650
	+	46	89		58	77		102	33	
diff-CA125	–	23	51	0.152	32	42	0.567	52	22	0.754
	+	31	42		35	38		53	20	
diff-CA724	–	24	50	0.669	26	48	0.275	50	24	0.223
	+	27	65		40	52		70	22	

*VOLI* Vascular or lymphatic invasion

## Discussion

Serum tumor markers are widely applied in diagnosis, therapeutic effect assessment and disease recurrence monitoring [9], and a series of studies have explored the diagnostic and prognostic value of various serum tumor markers in gastric cancer [10]. However, most of these studies were based on patients who had undergone surgery with or without adjuvant therapy, and only a few focused on patients with NCT in gastric cancer [11, 12].

Other studies have shown that, CA125 was associated with the R0 resection rate [13], recurrence, peritoneal dissemination [14] and OS in unresectable advanced or recurrent GC patients [15]; CA199 was related to pN [7, 16–18] and pTNM stage [17]; and CA724 was related to the pathological stage and had a good diagnostic value for gastric cancer [19]. However, none of the patients in those studies underwent NCT.

In our study, the levels of CA199, CA125 and CA724 before and after NCT could predict prognosis, and the changes of CA199 and CA125 were related to the outcome. Similar results had been presented by many studies based on GC patients without NCT [8, 13, 19, 20]. Nevertheless, we found that only CA724 was an independent prognostic factor in the multivariable analysis before and after NCT. It is noteworthy that this independent significance was lost in the diff- group, which was different from the findings of some studies. Zou et al. [11] claimed that the change in CA724 could reflect the therapeutic effect of NCT. Another paper suggested that a decrease in CA724 could lead to a better prognosis [12]. The difference in findings might be because more clinicopathological factors were included in the multivariable analysis in our study. Despite this, CA724 was still an independent predictive factor for GC patients who had undergone NCT.

Although CA724 had a high diagnostic value in GC, its sensitivity was only approximately 45.0% [19]. Moreover, in China, CA724 has been suggested to be associated with *Helicobacter pylori* infection [21] and even geographical environmental factors, such as temperature [22]. These findings imply that there might be a bias to evaluate the condition of patients merely depending on CA724. Much work has been done to address this problem. For example, TKI, an enzyme involved in the regulation of the mammalian cell cycle, was another choice of marker in gastric cancer [19]. Moreover, the combination of CA724, CA199, CA125 and CEA could also improve the diagnostic capability [9, 12, 19, 23]..

Regarding pathological factors, we found that CA199, CA125 and CA724 before and after NCT were all correlated with lymph node metastasis and ypTNM stage. In previous studies of GC patients who underwent curative gastrectomy, preoperative CA199 could predict lymph node metastasis [17, 18] and pTNM stage [17]. CA724 was associated with nodal involvement [9] and pathological stage in advanced gastric cancer patients [19]. To supplement those findings, we confirmed them in patients with preoperative therapy. In addition, we found that CA125 was also related to ypN and ypTNM stage.

In our study, the VOLI rate was significantly higher in the positive post-CA724 group, while in pre-CA724 group, the difference was not significant. However, Sun et al. [12] suggested that pre-CA724 was related to vascular invasion. This finding might suggest that CA724 could be used to assess lymphatic or vascular invasion, but more evidence is needed.

It was unexpected that CEA was not related to prognosis in our study. The reasons might include that a different cutoff value was used in our study, and NCT might influence the predictive value of the tumor marker. Although a team from Japan supported a similar result [7], a large number of studies suggested the opposite result [10, 16, 18, 24]. Nevertheless, in our study, CEA was related to CA199 and CA125, which was in line with other studies [7, 19]. It is noteworthy that these connections weakened after NCT, which might mean that preoperative treatment could blur these relationships.

The limitations still exist in this study. First, due to the nature of retrospective research, some patients did not have all values of the markers, which hindered the exploration of the combination of markers. Second, the sample size was not large enough. The sample numbers of AFU and AFP in every group were so small that they were not included in the further analysis. This limitation might also contribute to the reason why some *P* values were more than 0.05 but smaller than 0.1. Third, we used the optimal cutoff values derived from the Youden index, which disturbed the comparison with other studies.

Despite these limitations, our study still has some merits. Our sample size is relatively small, but we focused on a specific group of patients. The cutoff values of tumor markers used in our study were calculated by statistical methods, because there is lack of strong evidence on the optimal cutoff values, which might vary according to the therapy, tumor location, and even geographical features. We not only illuminated the prognostic significance of these tumor markers, but also confirmed their abilities to predict lymph node metastasis and pathological stage. These results are useful for assessing the condition of patients and making further clinical decisions.

## Conclusions

CA724 levels before and after neoadjuvant chemotherapy were both independent prognostic factors in GC patients undergoing NCT and curative surgery. Pre- and post-CA724 could also be used to predict lymph node metastasis and pathological stage. However, the change of CA724 due to preoperative treatment was not related to the prognosis in the multivariate analysis.

## Supplementary Information

**Additional file 1.** Number of patients with different tumor markers.

## Data Availability

The datasets used and/or analyzed during the current study are available from the corresponding author on reasonable request.

## Abbreviations

AFU: Alpha-l-fucosidase
AFP: Alpha-fetoprotein
CEA: Carcinoembryonic antigen
CA199: Carbohydrate antigen 19–9
CA125: Cancer antigen 125
CA724: Carbohydrate antigen 72–4
ypTNM: post-neoadjuvant therapy TNM stage
pTNM: pathological TNM stage
GC: Gastric cancer
NCT: Neoadjuvant therapy
OS: Overall survival
AJCC: American Joint Committee on Cancer
VOLI: Vascular or lymphatic invasion
NI: Nervous invasion
