# Supplementary material for: CA724 predicts overall survival in locally advanced gastric cancer patients with neoadjuvant chemotherapy
Source: BMC Cancer. 2021 Jan 5;21:4. doi: 10.1186/s12885-020-07666-8 (PMC7786973; doi:10.1186/s12885-020-07666-8)
Supplement: Supplementary file 1 — Additional file 1. Number of patients with different tumor markers. [file 12885_2020_7666_MOESM1_ESM.docx]

| Additional file 1 | Number of patients with different tumor markers | | | |
| --- | --- | --- | --- | --- |
| Tumor marker | No. of patients | |  |  |
|  | N- | N+ | Total | Percent |
| pre-AFU |  |  | 147 | 50.7% |
| >45.1 | 1 | 2 | 3 |  |
| ≤45.1 | 51 | 93 | 144 |  |
| pre-AFP |  |  | 152 | 52.4% |
| >2.6 | 20 | 49 | 69 |  |
| ≤2.6 | 33 | 50 | 83 |  |
| pre-CEA |  |  | 272 | 93.8% |
| >1.6 | 48 | 97 | 145 |  |
| ≤1.6 | 45 | 82 | 127 |  |
| pre-CA199 | |  | 274 | 94.5% |
| >24.9 | 15 | 54 | 69 |  |
| ≤24.9 | 80 | 125 | 205 |  |
| pre-CA125 | |  | 188 | 64.8% |
| >16 | 6 | 46 | 52 |  |
| ≤16 | 62 | 74 | 136 |  |
| pre-CA724 | |  | 224 | 77.2% |
| >4.6 | 18 | 65 | 83 |  |
| ≤4.6 | 49 | 92 | 141 |  |
| post-AFU |  |  | 101 | 34.8% |
| >40.9 | 7 | 15 | 22 |  |
| ≤40.9 | 27 | 52 | 79 |  |
| post-AFP |  |  | 102 | 35.2% |
| >4.6 | 10 | 35 | 45 |  |
| ≤4.6 | 25 | 32 | 57 |  |
| post-CEA |  |  | 243 | 83.8% |
| >3.3 | 24 | 50 | 74 |  |
| ≤3.3 | 60 | 109 | 169 |  |
| post-CA199 | |  | 243 | 83.8% |
| >62.6 | 3 | 26 | 29 |  |
| ≤62.6 | 81 | 133 | 214 |  |
| post-CA125 | |  | 172 | 59.3% |
| >11.2 | 22 | 62 | 84 |  |
| ≤11.2 | 44 | 44 | 88 |  |
| post-CA724 | |  | 187 | 64.5% |
| >5.9 | 17 | 56 | 73 |  |
| ≤5.9 | 45 | 69 | 114 |  |
| both-AFU | 24 | 54 | 78 | 26.9% |
| both-AFP | 24 | 55 | 79 | 27.2% |
| both-CEA | 80 | 150 | 230 | 79.3% |
| both-CA199 | 80 | 151 | 231 | 79.7% |
| both-CA125 | 54 | 93 | 147 | 50.7% |
| both-CA724 | 51 | 115 | 166 | 57.2% |
| pre-CEA+CA199+CA125+CA724 | 42 | 97 | 139 | 47.9% |
| post-CEA+CA199+CA125+CA724 | 44 | 77 | 121 | 41.7% |
| both-CEA+CA199+CA125+CA724 | 31 | 64 | 95 | 32.8% |
